# Transcript abundance on its own cannot be used to infer fluxes in central metabolism

**DOI:** 10.3389/fpls.2014.00668

**Published:** 2014-11-28

**Authors:** Jörg Schwender, Christina König, Matthias Klapperstück, Nicolas Heinzel, Eberhard Munz, Inga Hebbelmann, Jordan O. Hay, Peter Denolf, Stefanie De Bodt, Henning Redestig, Evelyne Caestecker, Peter M. Jakob, Ljudmilla Borisjuk, Hardy Rolletschek

**Affiliations:** ^1^Brookhaven National Laboratory, Department of Biological, Environmental and Climate SciencesUpton, NY, USA; ^2^Leibniz-Institut für Pflanzengenetik und KulturpflanzenforschungGatersleben, Germany; ^3^University of Würzburg, Institute of Experimental Physics 5Würzburg, Germany; ^4^Bayer CropScience NV, Trait ResearchZwijnaarde, Belgium

**Keywords:** *Brassica napus*, ^13^C-metabolic flux analysis, flux balance analysis, central metabolism, targeted metabolite profiling, lipid biosynthesis, oilseeds

## Abstract

An attempt has been made to define the extent to which metabolic flux in central plant metabolism is reflected by changes in the transcriptome and metabolome, based on an analysis of *in vitro* cultured immature embryos of two oilseed rape (*Brassica napus*) accessions which contrast for seed lipid accumulation. Metabolic flux analysis (MFA) was used to constrain a flux balance metabolic model which included 671 biochemical and transport reactions within the central metabolism. This highly confident flux information was eventually used for comparative analysis of flux vs. transcript (metabolite). Metabolite profiling succeeded in identifying 79 intermediates within the central metabolism, some of which differed quantitatively between the two accessions and displayed a significant shift corresponding to flux. An RNA-Seq based transcriptome analysis revealed a large number of genes which were differentially transcribed in the two accessions, including some enzymes/proteins active in major metabolic pathways. With a few exceptions, differential activity in the major pathways (glycolysis, TCA cycle, amino acid, and fatty acid synthesis) was not reflected in contrasting abundances of the relevant transcripts. The conclusion was that transcript abundance on its own cannot be used to infer metabolic activity/fluxes in central plant metabolism. This limitation needs to be borne in mind in evaluating transcriptome data and designing metabolic engineering experiments.

## Introduction

Gaining an understanding of metabolism is a central goal of biology, as it defines the growth and development of an organism. In the ‘omics era, cell processes can be described at the level of the transcriptome, the proteome or the metabolome, or possibly combinations thereof. Although none of these approaches actually measure metabolic activity, the data obtained can be used to make indirect inferences. The direct determination of metabolic flux would be highly desirable, as it represents a highly informative aspect of the metabolic phenotype (Schwender, 2008; Sweetlove et al., 2014). Metabolic flux analysis (MFA), which at present relies on the tracking of isotopically labeled material within a target metabolic network, has produced some unique insights into plant metabolism and its regulation (Rontein et al., 2002; Schwender et al., 2004; Spielbauer et al., 2006; Alonso et al., 2007; Williams et al., 2010; Allen and Young, 2013; Masakapalli et al., 2014). The approach is, however, too technically challenging for it to currently enjoy widespread use.

The ideal route to understanding metabolic phenotypes is to take a combined ‘omics approach, but for the most part this remains an aspirational rather than a realistic strategy. Instead, the common analytical basis for understanding metabolic activity is through the acquisition of enormous volumes of transcription data generated from microarrays and high throughput sequencing. An important question is therefore the extent to which the transcriptome does in fact reflect metabolic activity. In other words: is mRNA abundance at all relevant for flux control in central plant metabolism? Or, is it may be wise to restrict the use of transcriptomic data to the definition of metabolic capability, which may be developmental stage- and/or tissue-specific (Belmonte et al., 2013). The literature provides numerous examples of discordance between transcript, protein and metabolite abundance (Piques et al., 2009; Marmagne et al., 2010; Bourgis et al., 2011; Baerenfaller et al., 2012; Fernie and Stitt, 2012; Walley et al., 2013). A direct, large-scale comparison of relevant transcript abundance (encoding specific enzymes and metabolite transporters) with metabolic fluxes has yet to be made, especially in a plant system where this depth of information is currently not available.

Similar considerations apply to the outcome of proteomic or metabolomic studies. The proteome may define the enzymatic machinery, but it does not easily factor in the various post-translational modifications which are known to exert a major influence over the biological activity of many proteins (Oliveira et al., 2012). For example, most glycolytic and tricarboxylic acid cycle enzymes, as well as many metabolite translocators present in the seed, are phosphorylated and/or acetylated (Finkemeier et al., 2011; Meyer et al., 2012; Walley et al., 2013). The metabolome presents a better picture of the metabolic phenotype, but can only inform regarding steady state metabolite levels. At steady state, the fluxes of delivery and withdrawal are identical, and the metabolite level depends on thermodynamic equilibria/kinetic properties of the enzyme. Thus, metabolite abundance on its own is an insufficient basis for inferring metabolic activity.

The aim of the current study was to attempt to relate transcript abundance with metabolic flux on a genome-wide scale, with a view to assessing its relevance for understanding the central metabolism of a crop seed. The plant model employed was the developing embryo of oilseed rape (*Brassica napus*), an important temperate oilseed crop, and a close relative of the model plant *Arabidopsis thaliana*. Our data demonstrated a disconnect between transcript abundance and the corresponding metabolic flux. The seemingly low contribution of transcriptional regulation over metabolic flux control and the biological significance of this finding are discussed.

## Materials and methods

### Plant growth and *in vitro* culture

Plants of oilseed rape accessions 3170 and 3231 (both maintained by the IPK Genebank) were grown under conditions of 18°C, a 16 h photoperiod (400 μmol quanta m^−2^ s^−1^) and 60% relative humidity. Intact embryos were isolated 20 days post flowering and maintained for 10 days under 50 μmol quanta m^−2^ s^−1^ at 20°C in a liquid culture containing a source of organic carbon and nitrogen, as described by Schwender et al. (2006). Three independent batches of embryos were sampled from each accession. Following their *in vitro* culture, the embryos were snap-frozen, freeze-dried, weighed and pulverized. Note that the same system was used by Hay et al. (in press) in a companion paper to describe flux distribution and pathway usage in the central metabolism.

### Nuclear magnetic resonance based-imaging of lipid distribution

The MRI data were acquired on a 11.7 tesla AMX instrument (Bruker GmbH, Rheinstetten, Germany) using an absolute quantification method (Neuberger et al., 2009). In brief, the mature seeds of both accessions were placed into an NMR-glass tube and inserted into a home built 5-mm Helmholtz coil. A standard multi-slice multi-echo spin echo sequence with a CHESS suppression module on the water resonance was applied to acquire high-resolution lipid images of the seed. Furthermore, the bandwidths of the excitation and refocusing pulses were set to 2000 Hz and a global excitation was used in order to acquire lipid signal only. Before the imaging sequence, a T1 measurement of the lipid containing seeds was conducted to determine the repetition time (TR) necessary to avoid for T1 correction during the quantification process. As a result of this measurement (T1 ~500 ms; data not shown), the TR was chosen to be at least ~5^*^T1. Resolution of up to 39 μm isotropic could be achieved after 2 averages, 12 echoes, 1 repetition (TR 2500 ms; TE 7.5 ms) and total experimental time 5 h 40 min. To calculate the volumes of the different seed organs, the MRI-dataset was imported into the software AMIRA (Mercury Inc.) and image segmentation was conducted. The average signal in each of embryo organs was calculated as previously described (Borisjuk et al., 2013a).

### Metabolite and fatty acid analysis

Metabolic intermediates were extracted and measured by liquid chromatography coupled to mass spectrometry as detailed in the companion paper (Hay et al., in press). The identity of the various compounds was verified by comparison of their mass and retention time with those of authenticated standards. External calibration was applied for all compounds using these standards. Fatty acid composition was assessed by gas chromatography, following Borisjuk et al. (2013a).

### Flux analysis and metabolic modeling

Experimental details and the analysis of data related to MFA and flux variability analysis are detailed in the accompanying paper by Hay et al. (in press). In short, ^13^C- MFA was used to determine the flux distribution in central metabolism of the two rapeseed accessions. The flux information was transferred by flux ratio constraints into a large scale metabolic model, bna572+. By Flux Variability Analysis flux bounds were predicted for the 671 reactions in the network, confining the feasible optimal flux space for each of the two accessions. Since developing embryos differed mainly in lipid and starch content (lipid higher in 3170, starch higher in 3231), we designated a reaction “T” (triacylglycerol, TAG responsive) if the flux magnitude is higher in accession 3170, “S” (starch responsive) if the flux magnitude is higher in 3231, and “N” if a flux is unchanged. For a difference to be judged as a change the numerical tolerance was 10^−7^ μmol/h. bna572+ is referenced to the *Arabidopsis* genome, i.e., reactions have associations with *Arabidopsis* gene identifiers (Gene-Protein-Reaction associations). As the *B. napus* transcriptome was annotated by sequence homology to *Arabidopsis*, differences in transcript abundance between the two accessions could be compared to differences in flux based on the common *Arabidopsis* reference.

### Methods for transcriptome analysis

#### RNA extraction and RNA-sequencing

The total RNA from the *B. napus* embryos was isolated using the Spectrum Plant Total RNA kit (Sigma) according to the manufacturer's protocol. The samples for transcriptome analysis were prepared using an Illumina kit following the manufacturer's instructions. Briefly, beads with Oligo(dT) were used to isolate poly(A) mRNA from the total RNA. Fragmentation buffer was added in order to convert mRNA into short fragments. Taking these short fragments as templates, random hexamer-priming was used to synthesize the first-strand cDNA. The second-strand cDNA was synthesized using buffer, dNTPs, RNaseH, and DNA polymerase I. Short fragments were purified with the QiaQuick PCR extraction kit and resolved with EB buffer for end repair and for addition of poly(A). Subsequently, the short fragments were connected with sequencing adapters. Following agarose gel electrophoresis, the suitable fragment fraction was selected for PCR amplification. Finally, the library was sequenced using Illumina HiSeq™ 2000 (100bp, paired-end).

#### Transcription estimation

We assembled a draft transcriptome-wide collection of 222,331 (average length 1452 bp) putative cDNA sequences by aligning RNA-seq reads to the genome references of diploid progenitors *Brassica rapa* and *Brassica oleracea* using the splice-aware aligner TopHat (v2.0.8b) (Trapnell et al., 2009) and subsequently applying the Cufflinks (v 2.0.2) reference annotation based transcript assembly (Roberts et al., 2011). Reads that did not align to the reference genomes were assembled separately using Trinity (trinityrnaseqr20130814) (Grabherr et al., 2011). Gene expression estimation was subsequently done by using RSEM (Li and Dewey, 2011) which deconvolutes expression coming from very similar sequences such as homeologs. Expression estimates from splicing isoforms were summarized to a gene-level transcripts per million and inferred gene-specific read counts.

#### Differential transcription

The estimateSizeFactors and estimateDispersions functions in the DESeq package v1.12.1 (www.bioconductor.org) using default parameters were run for the normalization of the RNA-seq read count data. The negative binomial test implemented in DESeq was used to assess differential expression between the two genotypes. Variance stabilizing transformation (varianceStabilizingTransformation function in the vsn package v3.28.0 was performed on the normalized expression estimates.

#### Gene ontology (GO) analysis

To assign each putative differentially transcribed gene (DTG) to a particular functional category, GO annotations were retrieved from TAIR (https://www.arabidopsis.org/) using the org.At.tair.db v2.9.0 package (www.bioconductor.org). A Fisher exact test, as implemented in topGO v2.12.0 (www.bioconductor.org), was employed to test for the significance of category enrichment (Alexa et al., 2006). The “elim” algorithm, which de-correlates the GO graph topology by iteratively removing genes mapped to significant GO terms from more general GO terms, was used to increase interpretability of the results. Correction for multiple testing was done using the Benjamini and Hochberg correction implemented in the R function p.adjust (Benjamini and Hochberg, 1995).

#### Quantitative RT-PCR (qPCR)

A 2 μg aliquot of total RNA extracted from the embryo material was converted to single stranded cDNA using a RevertAid First Strand cDNA Synthesis kit (ThermoScientific, Germany). A 100 ng template of this cDNA provided the template for a qPCR based on the SYBR® Green PCR Master Mix (Invitrogen, Germany). The necessary primers, designed with Primer3 software (http://primer3.ut.ee) to generate an amplicon size of 150–200 bp, targeted mainly the 3′-UTR of the selected genes (three pairs of primers per gene). The specificity and amplification efficiency of each primer pair was checked by means of running standard curves with melting curves. Each assay comprised three biological replicates, each of which was repeated three times. Relative transcript abundances were estimated using the 2-ΔΔCt method (Livak and Schmittgen, 2001). The chosen reference sequence for normalization purposes was the housekeeping gene ubiquitin carrier protein 9 (UBC9). Means were statistically compared using the Students' *t*-test. Primer sequences used in qPCR are listed in Supplementary Table 1.

#### Data repository

RNASeq data derived from this study (accession SRP047515), including all project and biosample data were made publicly available under the following links:

http://www.ncbi.nlm.nih.gov/bioproject/?term=PRJNA262144

http://www.ncbi.nlm.nih.gov/biosample/?term=SRS709415

http://www.ncbi.nlm.nih.gov/biosample/?term=SRS717382.

### Data visualization using mapman and vanted software

The MapMan visualization tool (http://mapman.gabipd.org) was used for the functional characterization of DTGs. The threshold for significance was set to 0.01 with a minimum log2 (fold change) of 2. Differentially expressed transcripts that exhibited similarity to annotated *A. thaliana* genes, but showed a different trend in expression level were sorted out for further analysis with MapMan software.

The VANTED framework (Rohn et al., 2012) was used to visualize the conformance between flux and transcript data. The latter comprised the log2 (fold change) in transcript abundance between accessions 3231 and 3170. Transcripts were selected on the bases that their differential transcription was associated with a *p*-value of < 0.01, showing a significant change in transcript abundance, and were associated with bna572+ stoichiometric model reactions which showed a flux responsiveness for triacylglycerols (TAGs) as the major class of storage lipid. The flux data consist of flux variability bounds, calculated using the extended bna572+ stoichiometric model (for details, see the Hay et al., in press). To be considered as a change in flux, the threshold for the absolute flux response value was set to 10^−7^ μmol h^−1^. In addition, the only fluxes considered were those associated with a flux change for oil responsiveness. To show a change in flux, the slopes of the minimum and maximum flux of both accessions were estimated. Absolute values were used as the interest was exclusively in changes of the net flux. A mean value was then calculated from the two slope values; a positive net flux difference between the two accessions indicated an increase and a negative one a decrease in flux. This value is called flux-change-value. In order to indicate congruent changes in both transcript and flux for each reaction, a simple transformation was performed, which assigned a value of +1 where the change was congruent but −1 where it was not. A congruent change is defined as the situation where both transcript abundance (log2 fold change) and flux-change-value are positive in accession 3170 vs. 3231 or vice versa. VANTED was used to create a simple hierarchical map of the bna572+ model (Hay et al., in press), where reactions were grouped according to their pathway and cellular compartment, along with any transporters connecting compartments. Each node in this map was identified by a unique model-reaction identifier.

## Results

### Experimental strategy

**Figure 1:**
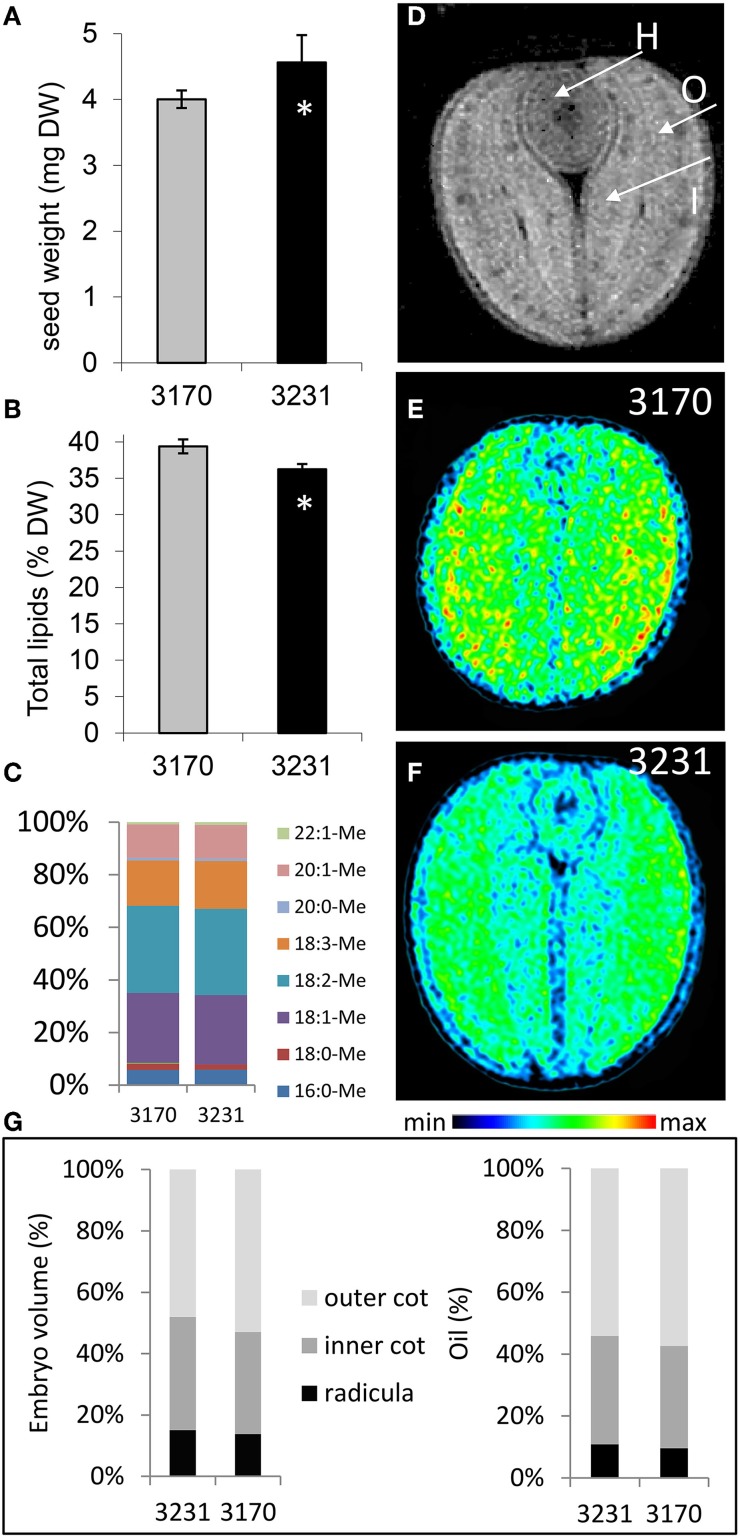
**The seed of oilseed rape accessions 3170 and 3231. (A)** Seed weight, **(B)** total lipid content (expressed as % of dry weight), **(C)** individual fatty acid profile (expressed as % of total fatty acids). **(D)** A cross-sectional MRI-based image showing the hypocotyl [H], the outer cotyledon [O] and the inner cotyledon [I]. MRI-based imaging of lipid distribution in the mature seed of accessions **(E)** 3170 and **(F)** 3231. Rainbow color scale indicates relative lipid concentration for images in **(E,F)**. **(G)** The relative contribution to both embryo volume and total stored lipid of the radicula, inner and outer cotyledon; calculation was based on MRI data. Bars in **(A,B)** represent the mean of 10 samples of four seeds. +/− indicates the standard deviation of the mean. The star (^*^) indicates statistical significance (*t*-test, *p* < 0.05).

Based on an initial screen to identify rapeseed accessions which contrasted with respect to their seed composition (http://documents.plant.wur.nl/cgn/pgr/brasedb/), we selected two accessions: 3170 accumulated more lipid but slightly less dry matter in its mature seed than 3231 (Figures 1A,B). The composition of fatty acids in their mature seed was almost identical (Figure 1C). By applying magnetic resonance imaging (MRI) we analyzed embryo structure and spatial oil accumulation pattern in mature seeds. Lipid distribution is given in Figures 1E,F and as three-dimensional models (Supplementary Video 1). Our analysis revealed that the lipid fraction elevated in accession 3170 vs. 3231 was due to rising concentrations throughout the embryo. Calculations based on the MRI data suggested that the relative contribution to both seed volume and total stored lipid of the radicula (=hypocotyl), inner and outer cotyledon was very similar for the two accessions (Figure 1G). Thus, the difference in lipid content between the accessions was likely related to an altered carbon partitioning rather than any structural/organ-specific effects (Borisjuk et al., 2013a).

For further work we used cultivated embryos, grown under standardized *in vitro* conditions (Schwender et al., 2006), as only this approach allows the application of ^13^C-MFA. In addition, we used the cultivated embryo material for targeted metabolomics (LC/MS) and genome wide transcript profiling (RNA-sequencing). As detailed in the Hay et al. (in press), the MFA was informative for 79 metabolic reactions with the central metabolism, and allowed the elaboration of a genome referenced flux balance metabolic model bna572+ which included 671 biochemical and transport reactions. This information was eventually used for comparative analysis of transcript vs. flux to answer one central question: What can transcriptomics (and metabolomics) tell us about metabolic phenotypes?

### Steady state metabolite levels

**Figure 2:**
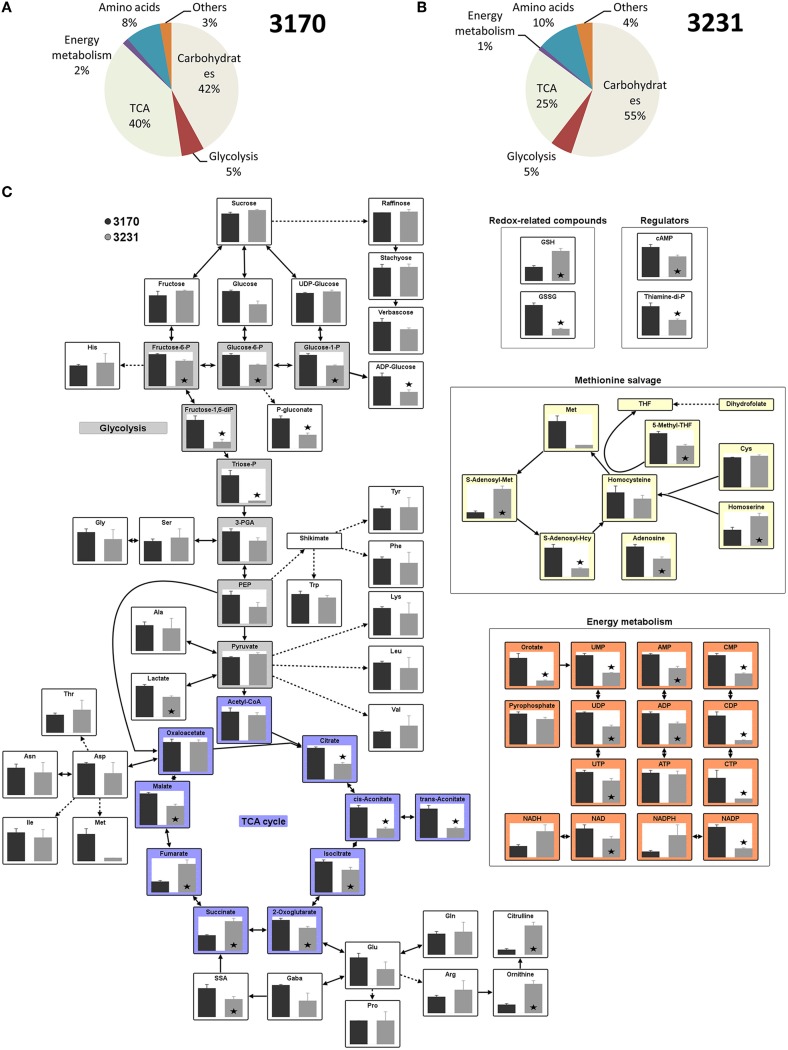
**Metabolites produced by *in vitro* cultured embryos of two oilseed rape accessions. (A,B)** The relative pool sizes across all metabolites in accession **(A)** 3170 and **(B)** 3231. **(C)** Steady state metabolite levels of the high lipid accumulator accession 3170 (black bar) and the low lipid accumulator accession 3231 (gray bar), as measured by liquid chromatography/mass spectrometry. The standard error was calculated from measurements taken from five technical replicates per each of three biological replicates. Asterisks indicate means differing significantly (*p* < 0.05, *t*-test) between accessions.

The use of the liquid chromatography/mass spectrometry platform enabled the quantification of 79 metabolite intermediates present in the embryo central metabolism (listed in Supplementary Table 2). In both accessions, soluble sugars (mostly sucrose) represented the major fraction (on a mol g^−1^ dry weight basis), followed by organic acids and free amino acids (Figures 2A,B). Glycolytic intermediates, nucleotides, cofactors, redox-related intermediates (glutathione, ascorbate), along with a few other possible signaling compounds, represented only ~10% of the total metabolite pool. The two accessions had quite distinct metabolic profiles (Figure 2C). High-oil accession 3170 revealed a marked increase in the content of hexose phosphates and some glycolytic intermediates. With respect to each of the three redox couples (reduced/oxidized glutathione [GSH/GSSG], NADH/NAD and NADPH/NADP), the oxidized intermediate was more abundant in accession 3170 than in 3231. Differences were also noted for the abundance of the nucleotides AMP, UMP, and CMP (as well as for their di- and tri-phosphates), indicating a shift in energy metabolism. Most of the common organic acids (malate, citrate, isocitrate, cis-/trans-aconitate and oxoglutarate) were more abundant in high-oil accession, while succinate and fumarate were less well represented. This pattern suggested inter-accession differences in the activity of the tricarboxylic acid (TCA) cycle or in some of its individual steps. The levels of all of the highly abundant sugars (sucrose, hexoses) and free amino acids (Ala, Gln, Glu, Asn, Pro) were indistinguishable between the accessions.

### Metabolite abundance in relation to metabolic flux

**Figure 3:**
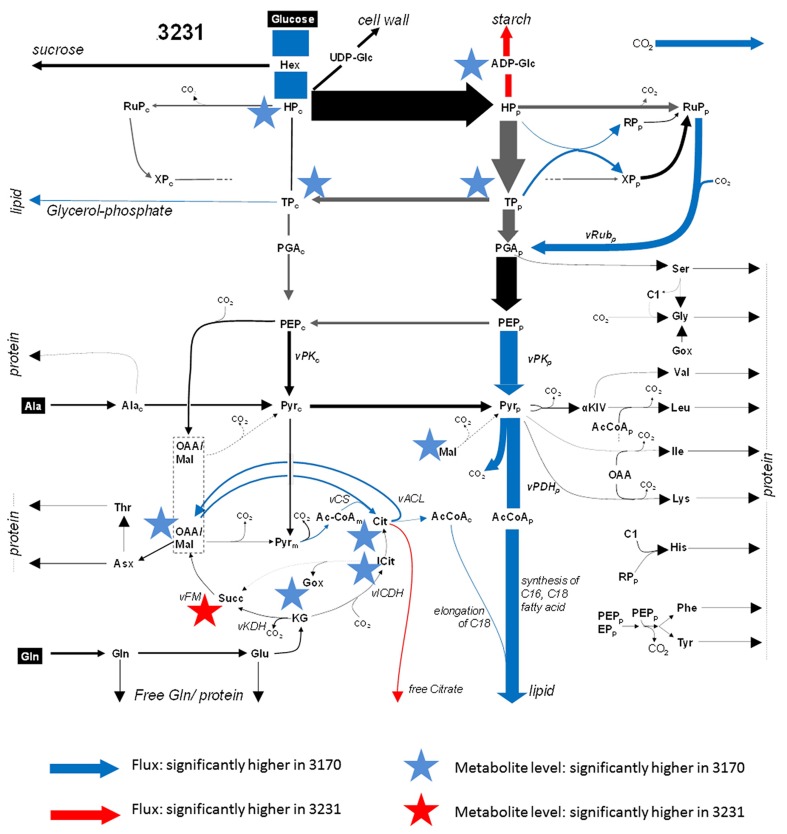
**Combined flux map and significant changes in metabolite steady state levels within primary metabolism**. A flux distribution map of glycolysis, the TCA cycle and amino acid synthesis for accession 3231. Arrows indicate flux and the asterisks indicate metabolite levels. Significant higher (lower) values in accession 3170 are marked in blue (red). The thickness of each arrow corresponds to the flux value. Gray arrows denote high statistical uncertainty in flux. The flux map was taken from the companion paper by Hay et al. (in press).

In order to compare metabolite profiles with net flux rates (the latter taken from the Hay et al., in press, and listed in Supplementary Table 3), metabolite levels were pooled where the relevant reactions had been combined to form a single flux value; for example, the hexose phosphates represented an aggregate of glucose-6P, glucose-1P, fructose-6P, and fructose-1,6-diP. Note that for some of the reactions, the flux analysis was able to distinguish cellular compartment-specific branches (e.g., plastidial vs. cytosolic glycolysis), but the metabolite analysis was unable to make this distinction. Since there is absolutely no expectation that the subcellular concentration of a metabolite will be the same, we did not attempt to apply statistical (correlation) tests, but rather looked if some significant differences in net flux (^13^C-MFA dataset) between the accessions were accompanied by obvious shifts in the corresponding intermediates. The outcome of the combined analysis is presented in Figure 3. Less than half of reactions for which significant differences in flux between the accessions were identified also displayed a significant shift in the concentration of either the relevant substrate or reaction product. For example, the conversion of ADP-glucose to starch was higher in low-oil accession 3231, and ADP-glucose was depleted. The high-oil accession exhibited a higher flux in the phosphorylation of hexose, which corresponded to the accumulation of hexose phosphates. While the glycolytic flux of triose-P [TP] to P-glycerate [PGA] did not differ among accessions, we detected a significant difference of substrate TP. Further downstream in glycolysis, differences in flux induced no detectable differences in metabolite intermediate levels. The fluxes associated with a number of reactions linked to the TCA cycle were enhanced in high-oil accession, matched by a higher content of most of the TCA cycle intermediates (except succinate). Flux associated with the synthesis of storage lipids (the trait which distinguished the two accessions) was, however, not reflected in the size of the acetyl-CoA pool. Consistent with the lack of any significant difference between the accessions with respect to the level of amino acids present, the flux model did not feature any major difference in the synthesis of storage proteins.

### Transcriptome comparison

To obtain a global transcriptomic map of cultured embryos, we applied RNA-sequencing (RNA-Seq) to the two rapeseed accessions (three biological replicates each); the dataset is listed in Supplementary Table 4A. The qPCR validiation of the RNA-Seq-based identification of differentially transcribed genes (DTGs) featured eight transcripts. Although the estimates of transcript abundance derived from the two analytical platforms were not fully concordant, the direction of differential transcription was confirmed in each case (Supplementary Figure 1A) and the correlation coefficient between the two estimates across all eight genes was 0.96.

Based on the definition of differential gene expression (padj<0.01 and more than 2-fold change; see Materials and Methods), the RNA-Seq data suggested that 2156 transcripts were more abundant in accession 3170 than in 3231 (up-regulated DTGs), while 1958 were less abundant (down-regulated DTGs) (Supplementary Table 3A). The DTGs were assignable to a number of functional GO categories, including some represented in the major pathways operating in the central metabolism, such as “glycolysis,” “photorespiration,” “photosynthetic electron transport” and “gluconeogenesis” (Supplementary Table 4B).

Given that embryos of the two accessions differed mainly in lipid (higher in 3170) and starch (higher in 3231) content and in analogy to the flux responsiveness types “T” (TAG responsive, i.e., flux increasing with higher lipid content in 3170) and “S” (starch responsive, i.e., flux increasing with higher starch content in 3231), we designated significantly higher transcript levels as “T” and “S,” respectively. For further interpretation the genome of the closely related model plant species *Arabidopsis thaliana* was used as a reference. All transcript contigs were annotated against the *Arabidopsis* proteome based on sequence similarity (highest BLASTX score, *E*-value cut-off < 10^−10^). Supplementary Table 3A lists information aggregated from 89315 transcripts into 19087 *Arabidopsis* associated gene identifiers. *Arabidopsis* related MapMan ontology is also shown (Thimm et al., 2004; TAIR10 based ontology). Table 1 summarizes statistics on transcript-, gene- and reaction level significance in differential expression. In average, each gene ID was associated with 4.7 *B. napus* transcripts. The transcript-level significance calls were integrated in a most stringent way, by defining conflicting calls on the transcript level as altogether non-significant on the gene level. This way, there were 1064 gene level “T” calls and 911 “S” calls (Table 1). Furthermore, the normalized transcript read counts were summed on the gene level for both accessions (Supplementary Table 3A). For 847 of gene-level “T” calls, the summed expression was higher in high-oil accession, i.e., significance calls and the ratio of summed read counts agreed. In similar, for 717 gene-level “S” calls there was agreement with the ratio of summed expression values (Supplementary Table 3A).

**Table 1:** **Statistics of differential transcription calls (padj < 0.01 and more than 2-fold change) and aggregation of transcript level significance calls into genes (annotation by *Arabidopsis* genes) and reactions**.

**DIFFERENTIAL EXPRESSION IN *B. NAPUS* TRANSCRIPTS (3170 vs. 3231)**	
Number of transcripts annotated by homology to *Arabidopsis* genes	89315
Number of associated *Arabidopsis* gene identifiers	19087
Average number of transcripts per *Arabidopsis* gene identifier	4.7
Number of transcripts with significantly higher expression in 3170 (“T”)	2165
Number of transcripts with significantly higher expression in 3231 (“S”)	1958
**AGGREGATED GENE-LEVEL SIGNIFICANCE**	
Transcript level calls unambiguously combine to “T”	1064
+ ratio of summed expression values is higher in 3170	847
Transcript level calls unambiguously combine to “S”	911
+ ratio of summed expression values is higher in 3231	717
**AGGREGATED REACTION-LEVEL SIGNIFICANCE**	
Number of bna572+ associated genes for which expression was found	860
Reaction level calls unambiguously combine to “T”	45
+ flux higher in 3170	23
Reaction level calls unambiguously combine to “S”	82
+ flux higher in 3231	4

*See also Supplementary Tables 3A,B. “T”: up-regulated in accession 3170, “S”: up-regulated in accession 3231*.

Next, we used the MapMan ontology for *Arabidopsis* genes (Thimm et al., 2004) to classify the gene-level DE calls (Table 2). For most functional MapMan categories there where similar numbers of gene-level “T” and “S” calls, i.e., unambiguous up- or down regulation might occur in sub-BINS but is not resolved. A few categories for which the “S” and “T” calls differed by more than two-fold are highlighted in Table 2. Predominantly up-regulated in the high oil accession (“T”) are photosynthesis (PS), mitochondrial electron transport/ATP synthesis, and redox related genes (Table 2). Predominant up-regulation in accession 3231 (“S”) was found in the categories cell wall, major CHO metabolism (starch synthesis, starch degradation), and the oxidative pentose phosphate pathway (OPP).

**Table 2:** **Gene ontology for genes with differential transcript abundance**.

**BIN code**	**“S”**	**“T”**	“T”/”S”^*^	**MapMan classification**
35	197	232	1.18	Not assigned, no ontology/unknown^*^
29	114	142	1.25	Protein, aa activation/synthesis/targeting/postranslational modification/degradation/folding/glycosylation/assembly and cofactor ligation
27	77	91	1.18	RNA, processing/transcription/regulation of transcription/RNA binding/
26	31	37	1.19	Misc
34	30	37	1.23	Transport, p- and v-ATPases/metal/peptides and oligopeptides/unspecified cations/potassium/ABC transporters and multidrug resistance systems/unspecified anions/Major Intrinsic
				Proteins/sugars/porins/cyclic nucleotide or calcium regulated channels/amino acids/H+ transporting pyrophosphatase/ammonium/sulfate/metabolite transporters at the envelope membrane/metabolite transporters at the mitochondrial membrane/misc
20	31	35	1.13	Stress, biotic/abiotic
30	27	37	1.37	Signaling, in sugar and nutrient physiology/light/receptor kinase/calcium/phosphinositides/G-proteins/MAP kinases/14-3-3 proteins/misc/lipis/
31	30	25	0.83	Cell, organization/division/cycle/vesicle transport
28	31	19	0.61	DNA, synthesis/repair/unspecified
**1**	**9**	**29**	**3.22**	**PS, lightreaction/photorespiration/calvin cycle**
33	16	22	1.38	Development, storage proteins/late embryogenesis abundant/squamosa promoter binding like (SPL)/unspecified
11	15	18	1.20	Lipid metabolism, FA synthesis and FA elongation/glycolipid synthesis/FA desaturation/Phospholipid synthesis/TAG synthesis/lipid transfer proteins/unassigned/exotcs/lipid degradation
13	13	19	1.46	Amino acid metabolism, synthesis/degradation
**10**	**22**	**7**	**0.32**	**Cell wall, cellulose synthesis/hemicellulose synthesis/call wall proteins/degradation/modification/pectin esterases**
16	10	19	1.90	Secondary metabolism, isoprenoids/phenylpropanoids/N misc.alkaloid-like/sulfur-containing/flavonoids
17	12	10	0.83	Hormone metabolism, abscisic acid/auxin/brassinosteroid/cytokinin/gibberelin/salicylic acid
23	9	9	1.00	Nucleotide metabolism, synthesis/degradation/salvage/phosphotransfer and pyrophosphatases/deoxynucleotide metabolism
**9**	**1**	**15**	**15.0**	**Mitochondrial electron transport / ATP synthesis, NADH-DH/electron transfer flavoprotein/cytochrome c reductase/cytochrome c/cytochrome c oxidase/F1-ATPase**
3	7	7	1.00	Minor CHO metabolism, others/raffinose family/trehalose/myo-inositol/callose/sugar kinases/galactose
**21**	**4**	**10**	**2.50**	**Redox, thioredoxin/ascorbate/glutaredoxins**
8	5	7	1.40	TCA / org transformation, TCA/other organic acid transformatons
4	4	5	1.25	Glycolysis, cytosolic branch/plastid branch
**2**	**4**	**1**	**0.25**	**Major CHO metabolism, synthesis.starch/ degradation.starch**
18	3	2	0.67	Co-factor and vitamine metabolism, molybdenum cofactor/lipoic acid/riboflavin/folate
19	3	2	0.67	Tetrapyrrole synthesis, glu-tRNA synthetase/protochlorophyllide reductase/heme oxygenase/regulation/unspecified
**7**	**3**	**1**	**0.33**	**OPP, oxidative PP**
5	1	2	2.00	Fermentation, aldehyde dehydrogenase/PDC
12	2	1	0.50	N-metabolism, ammonia metabolism/N-degradation/misc
15	1	2	2.00	Metal handling, acquisition/binding
14	1	1	1.00	S-assimilation, AKN/APR
24	1	1	1.00	Biodegradation of Xenobiotics
32	0	2	Inf	Micro RNA, natural antisense etc
6	1	0	0.00	Gluconeogenesis / glyoxylate cycle.Malate DH
22	1	0	0.00	Polyamine metabolism
25	1	0	0.00	C1-metabolism

For each MapMan category (BIN) the number of genes up-regulated in accession 3170 (“T”) and up-regulated in accession 3231 (“S”) is given.

* (number of T-calls)/(number of “S” calls); ratio<0.5 and ratio>2 is indicated in bold. See also Supplementary Table 3A. ^*^BIN classification, 2nd level sub-BIN's of a gene set are listed separated by “/”).

**Figure 4:**
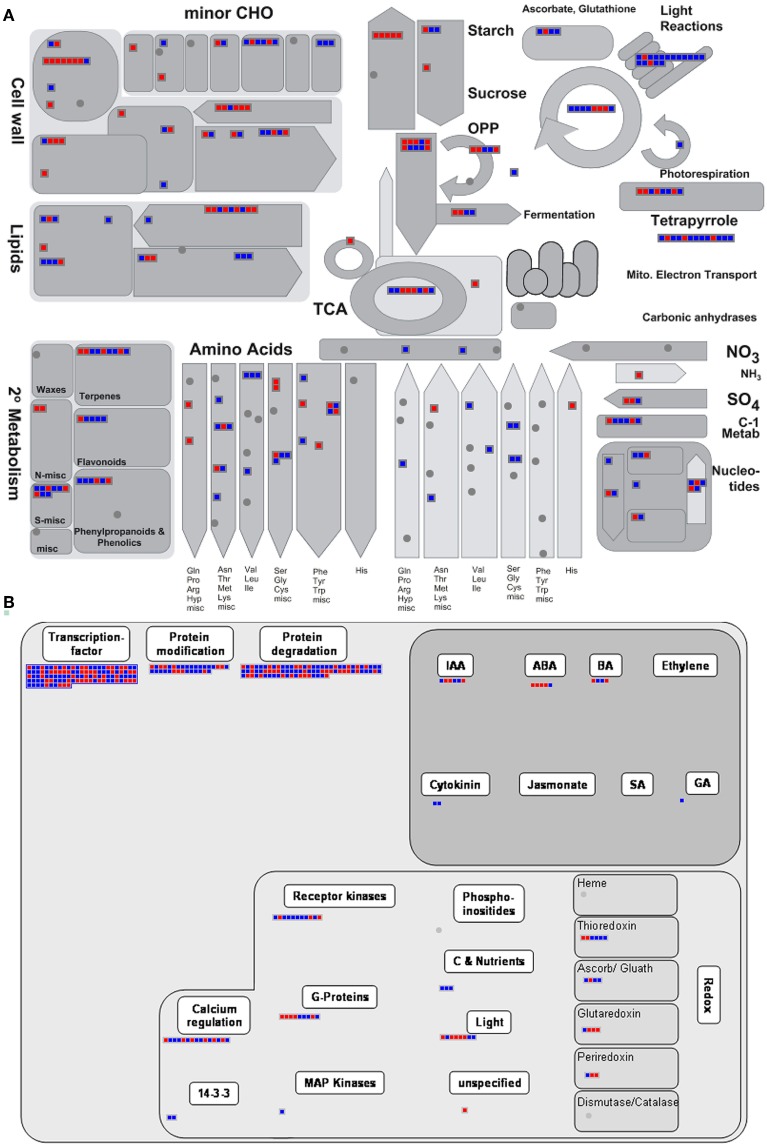
**Functional analysis of DTGs in the comparison between accessions 3170 and 3231**. Changes in transcript abundances of genes associated with **(A)** primary metabolism and **(B)** regulation as generated by MapMan software. Blue (red) denotes transcripts more (less) abundant in accession 3170.

MapMan was further used as tool for visualization. DTGs related to primary metabolism are shown in Figure 4A. Transcriptional differences were clearly visible for genes involved in lipid synthesis, photosynthesis, glycolysis, TCA cycle, amino acid synthesis and more. Up-regulated DTGs involved in lipid synthesis/storage included those encoding acetyl-CoA carboxylase (the first committed step in plastidial fatty acid synthesis), few enzymes involved in subsequent steps in fatty acid synthesis, oleosin 4 (a hydrophobic protein deposited on the surface of the oilbodies) and lipid transfer protein 2. As can be seen from Figure 4A, there was no clear evidence for the activation of the entire pathway controlling fatty acid synthesis. Photosynthesis-related DTGs are summarized in Supplementary Figure 2, where transcript abundance in high-oil accession was higher for genes encoding chlorophyll binding protein, various subunits of photosystem I and II, electron transporters and several enzymes within the Calvin cycle. Within the category energy metabolism (Supplementary Figure 3), up-regulated DTGs encoded fumarase1, succinyl-CoA ligase, pyruvate dehydrogenase E1 α and several components of the electron transport chain complex. With respect to the upstream pathway of glycolysis, there were several up-regulated DTGs encoding glycolytic enzymes, such as phosphoenolpyruvate carboxylase kinase, cytosolic glucose-6-phosphate isomerase, phosphoglucomutase and phosphoglucosamine mutase, while the down-regulated DTGs in this category included those encoding UDP-glucose pyrophosphorylase, phosphoglucomutase, phosphoglyceromutase, UDP-glucose pyrophosphorylase and phosphoglygerate/bisphosphoglycerate mutase. Thus, it appeared that only specific steps in the glycolytic pathway (rather than the pathway in its entirety) were differentially regulated in the high lipid accumulating accession.

The presence of DTGs within the MAPMAN category “regulation” provided some leads to explain the contrasting metabolic phenotypes of the accessions (Figure 4B). There were numerous DTGs encoding components of protein modification/degradation, receptor kinases, G-proteins and calcium regulators, which all suggest differences between the accessions at the level of post-transcriptional regulation and signal transduction. The abundance of 110 transcription factor transcripts varied between the two accessions. The transcription factor *Wri1* has been identified as a major controlling agent over the transcription of genes involved in fatty acid and storage lipid synthesis (Baud et al., 2009; Maeo et al., 2009). A *Wri1*-like sequence was about 32 fold more abundant in high-oil accession. Nevertheless, this sequence did not fall into the positive DTG category because its absolute abundance was too variable and rather low. When its transcription level was checked using qPCR, it was however confirmed to be up-regulated in high-oil accession (Supplementary Figure 1B). The functional significance of *Wri1* up-regulation remained obscure, as several of its potential targets were up-regulated (At3g02630, At4g01900, At5g46290, At5g15530, At1g01090, At2g38530) while others were down-regulated (At5g16390, At5g16240, At2g30200, At5g40610) in high-oil accession.

### Correlation of differential transcription with flux response

In the bna572+ metabolic model, 966 *Arabidopsis* genes map to 545 reactions. 860 of these genes where found in the *B. napus* transcript annotations. Supplementary Table 3B merges reaction level information from simulation of the two accessions in bna572+ with reaction level information on differential transcription. The gene-level significance calls were further integrated with highest stringency, i.e., by defining conflicting calls on the gene level as altogether non-significant on the reaction level. Accordingly, 45 and 82 reactions were called “T” and “S,” respectively (Table 1). Finally, 23 reaction level “T” calls coincide with unambiguous “T” responsiveness in the flux data. Only 5 reaction level “S” calls coincide with “S” responsiveness (Table 3). According to our data, reaction #95 (H_PYR_sym_c_p) is up-regulated in the high-oil accession, which agrees with increased pyruvate import into the plastid (flux is TAG responsive). Since low-oil accession has higher levels of starch, the up-regulation of reaction #344 (starchsynth_p) makes sense. In similar to the genes associated in bna572+ to the starch synthesis reaction, the “S” calls under MapMan classification 2 (Table 2) also indicates up-regulation of starch in low-oil accession.

**Table 3:** **Twenty seven reactions for which change in gene expression correlates with difference in flux between accessions 3170 and 3231**.

**index**	**Abbreviation; name**	**Lower/upper flux bound responsiveness**	**Reaction level differential expression**
26	PFK_c;phosphofructokinase	N;S	(S)()()()()^*^
55	RPI_p;ribose-5-phosphate isomerase	T;T	(T)()
93	PiC_c_m;phosphate carrier	T;T	()()(T)
95	H_PYR_sym_c_p;pyruvate transport	T;T	(T)
252	ADEK_c;adenosine kinase	T;T	()(T)
255	SHMT_c;glycine hydroxymethyltransferase	T;T	()()(X)()()(TT)
284	ABUTsynth_p;acetolactate synthase	T;T	(T)()()
294	IPMDY_p;3-isopropylmalate dehydratase	T;T	(TT)()(X)(T)
295	IPMD_p;3-isopropylmalate dehydrogenase	T;T	(X)(T)()
312	ACLACsynth_p;acetolactate synthase	T;T	(T)()()
318	ASUCS_p;adenylosuccinate synthase	T;T	(T)
320	IMPsynth_p;IMP cyclohydrolase	T;T	(T)
333	Phesynth_p;arogenate dehydratase	T;T	()()()()(X)(TT)
335	HOM_p;homoserine dehydrogenase	T;T	()(TT)
337	AK_p;aspartate kinase	T;T	()()(TT)()()
340	DHS_p;3-deoxy-7-phosphoheptulonate synthase	T;T	(TT)()()
344	starchsynth_p;starch synthase	S;S	()()(S)(SSS)(SS)()()()()
348	PURH_p;phosphoribosylaminoimidazolecarboxamide formyltransferase	T;T	(T)
355	Glysynth_p;glycine hydroxymethyltransferase	T;T	()()(X)()(T)()(TT)
357	IGLPS_p;indole-3-glycerol-phosphate synthase	T;T	()(T)
361	Thrsynth_p;threonine synthase	T;T	()(TT)
456	SUCLA_m;Succinate-CoA ligase (ADP-forming)	T;T	()(T)()
458	GDC2_m;aminomethyltransferase	S;S	(S)
499	ACCase_c;acetyl-CoA carboxylase	T;T	(T)
541	PDHa_m;dihydrolipoyllysine-residue acetyltransferase	S;S	(S)(S)()
576	CMK_p;4-diphosphocytidyl-2-C-methyl-D-erythritol kinase 1	T;T	(T)
621	DHCRed_c;7-dehydrocholesterol reductase	T;T	(TTTT)

Flux Variability Analysis for 3170 and 3231 (loopless algorithm) using flux ratios and flux constraints derived from ^13^C-Metabolic Flux Analysis.

* *transcript level significance calls are aggregated at the gene level in parentheses. “(X)” means that no associated transcripts were found for an Arabidopsis gene identifier in bna572+. See also Supplementary Table 3B*.

The generic photon uptake flux in reaction #6 (Ex_ph_t; not associated to any gene) is found TAG responsive. Note that the embryo culture experiment of the two accessions took place in parallel under the same light conditions. However, this observation might be explainable considering the indication of up-regulation of photosynthetic genes in the high oil accession (Table 2). This suggests that high oil accession have more abundant components of the photosynthetic apparatus, in particular light harvesting and electron transport. Of the 29 cases of gene “T” upregulation for MapMan category PS (Table 2), 22 are under sub-category “lightreaction.” Up-regulation of the OPP might be associated with the low oil phenotype in accession 3231 (Table 2). Half of the altogether 27 reactions listed in Table 3 (284, 294, 295, 312, 318, 320, 333, 335, 337, 340, 348, 355, 357, 361) are TAG responsive and associated to the biosynthesis of 7 amino acids (Val, Leu, Ile, Trp, Phe, Tyr, His). In the bna572+ model, these amino acid biosynthesis reactions are stoichiometrically coupled to protein synthesis reactions. According to the biomass constraints (see Table 1 in the accompanying paper Hay et al., in press) the protein content in model 3170 is only slightly higher than for 3231.

### Visualization of transcript/flux couples show largely discordant pattern

**Figure 5:**
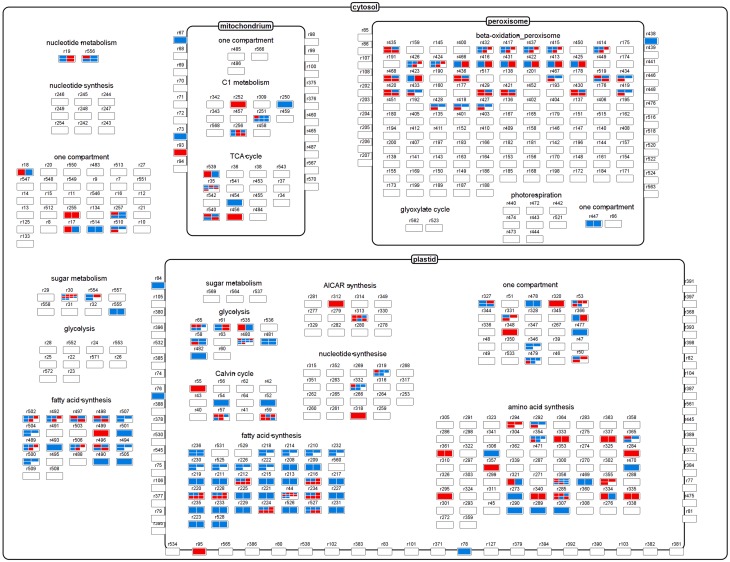
**The central metabolism, showing relevant reactions and transporters as nodes**. The heatmaps within each node correspond to differentially transcribed Brassica transcript IDs encoding enzymes/proteins related to the node's reaction. Red elements indicate a congruent fold change in both flux and transcript abundance, while blue ones indicate an opposed fold change.

The metabolic network derived from the bna572+ model comprised 515 nodes, with some model reactions for metabolite uptake and secondary pathways having been excluded. This set of reactions is shown as Figure 5, where reactions have been grouped on the basis of categories and cellular compartments in which they occur. Blue and red heat maps indicate significant differentially expressed transcripts, with change in transcript abundance being either in disagreement or agreement with a change in flux magnitude, respectively. Thus, red elements indicate that the changes in both flux and transcript abundance were congruent (in the same direction), while blue ones indicate changes in the opposite direction. About a quarter of the nodes contain DTGs, and are associated with several central metabolism pathways including glycolysis, the TCA and Calvin cycles, and fatty acid synthesis. The outcome of the analysis is that no single category shows a congruent fold change, rather there was a mixture of congruent and opposed changes in transcript abundance vs. flux changes. There were numerous examples where multiple transcript IDs, all encoding the same protein/enzyme, behave in an opposed way [examples are reaction nodes r30 (sucrose synthase) and r35 (mitochondrial malate dehydrogenase)]. The majority of metabolic reactions related to plastidial fatty acid synthesis showed opposed fold changes (as indicated by blue color in Figure 5), i.e., higher metabolic flux in high-oil accession but lower transcript abundance of the respective genes encoding the enzyme proteins involved in this pathway. The majority of metabolite transport reactions, mediating the exchange of substrates between subcellular compartments, did not show differential transcription (despite of changes in flux in several cases), and thus the corresponding nodes appeared empty. For one transport reaction (r93: mitochondrial Pi carrier) we found congruent change in both transcript abundance and flux, but for several other ones opposed changes (r67: mitochondrial oxaloacetate/succinate antiporter; r73: mitochondrial malate/oxaloacetate antiporter; r76: plastidial glutamate/malater antiporter; 84: plastidial glucose-6-P/Pi antiporter). All of the respective transporter-mediated fluxes were elevated in the high-oil accession.

Overall, there is little evidence for a scenario where a positive fold change of one data domain (transcript) is leading to a corresponding fold change in the related data domain (flux). The inevitable conclusion was that transcript abundance on its own cannot be used to infer metabolic activity/fluxes.

For further data exploration, the data mapping file used to generate Figure 5 has been provided in Supplementary Material File 1 (Mapping_transcript_vs._flux.gml). After installing VANTED software (http://sourceforge.net/projects/vanted/), the ViewData-Addon can be readily installed, using the addon manager implemented in VANTED—this feature will enable the user to browse details of the data contained within each node. For example, it allows to easily explore multiple mappings by listing all of the *Brassica* transcript IDs that are related to one model reaction ID (Supplementary Figure 4A). We also provide a mapping file (Supplementary Material File 2: frame.gml), which in addition to the data in Figure 5 provides information on the direction of the fold change of the flux values: red frame color indicates an increase of flux in the high-oil accession and a blue frame color a decrease of flux in accession. Such representation can further support data exploration as exemplified with the node element “r84” (Supplementary Figure 4B). This node represents the plastidial glucose-6-P/Pi antiporter. The mapping shows an opposed fold change of transcript abundance and flux, and the red frame color indicates an increased activity of this transport reaction in the high-oil accession (vs. 3231). The latter would be expected because the encoding genes (GPT1 [AT5G54800] and GPT2 [AT1G61800]) are known targets of the transcription factor *Wri1*, which generally favors oil storage.

## Discussion

Flux control in the central plant metabolism can be achieved by transcriptional regulation, among other ways. The aim of this study was to explore the correlation between metabolic flux and transcription on a genome-wide basis. The starting point was to select a pair of oilseed rape accessions contrasting in seed lipid accumulation: the mature seed lipid content of accession 3170 differs by some 3% from that of accession 3231 (Figure 1). This feature is also found in embryos cultured *in vitro* (see companion paper by Hay et al., in press). As the *in vitro* system enabled a steady state ^13^C-MFA, it provided an ideal platform for exploring the fluxome to provide the data necessary to compare with the more readily available transcriptomic and metabolomic data. A further advantage of the *in vitro* system was that it allowed the materials to be exposed to a well-controlled and identical growing environment, thereby avoiding the complication of interference due to environmental variation, while the use of embryos isolated from the seed coat/endosperm excluded any differential influence of maternal genotype.

The quantification of metabolites showed that the relative contribution of the dominant intermediates was rather similar between the two accessions, while some of the minor components (related to redox and energy metabolism) were quite distinct (Figure 2). The comparison of flux vs. metabolite level indicated that only for the minority of the reactions where flux changes were evident was a corresponding shift observable in the level of the relevant enzyme substrate or reaction end product (Figure 3). One obvious reason why this level of correlation was so low may be that it was not technically possible to define the sub-cellular distribution of metabolites. For example, a relationship between the pool size of acetyl-CoA (the precursor of fatty acid synthesis) and flux into fatty acids/TAG was expected (Rolletschek et al., 2005a,b), but not observed. The lack of correlation could be explained if it were the case that the plastidial levels of acetyl-CoA were rather low (with most of the analyte being held in the cytosol). It has been noticed that even modest changes in flux can markedly alter metabolite abundance (Williams et al., 2008), simply because the levels of most metabolites present represent only a very small fraction of the biomass. The conclusion is that the detection of metabolite abundance in the seeds of different oilseed rape accessions (and possibly the same applies for other species/tissues) using standard analytical techniques does not permit easily identifiable changes/perturbations in the flux map.

The transcriptomic analysis identified a few thousand putative DTGs, many active in the central metabolism. Some encoded TCA cycle enzymes, which chimed with the observed changes in both metabolite abundance and metabolic flux (Figure 3). The transcriptional activation of mitochondrial pyruvate dehydrogenase in high-oil accession may help to stimulate the production of acetyl-CoA and thereby encourage the elongation of fatty acids in the cytosol (Schwender and Hay, 2012), and so eventually enhance the deposition of lipids in the developing seed (Marillia et al., 2003). Additionally, a stimulated ATP/NADH synthesis may be needed by high-oil accession to support its higher rate of lipid synthesis, a process having a much higher energy demand than starch synthesis (which is more active in accession 3231). A further observation was the higher abundance of photosynthesis-related gene transcripts, which may help to increase the rate of CO_2_ fixation, to provide additional ATP/NADH and eventually to contribute to the light-dependent regulation of fatty acid synthesis (Borisjuk et al., 2013a). Notably, differential transcription analysis identified several genes related to the category “redox, thioredoxin/ascorbate/glutaredoxins” (Table 2) which coincided with significant changes in redox couples (Figure 2). It is tempting to speculate that the more oxidized state in high-oil accession reflects the higher demand for reducing equivalents in the high-oil phenotype.

Apart from DTGs' direct effect on metabolic activity, a large number of transcripts were involved in post-transcriptional regulation/signal transduction (Figure 4B). Some of these genes are associated with some very fundamental physiological processes, such as cellular differentiation, cell elongation, and protein turnover. Such processes can substantially affect local oil accumulation in the developing oilseed rape embryo (Borisjuk et al., 2013b), and thus may contribute materially to the difference between the high and low lipid accumulator phenotype observed in this study.

The glycolytic pathway, regarded as the heart of central metabolism, since it provides precursors and supplies a number of synthetic processes, provided several examples of opposed transcriptional regulation of certain enzymatic steps. Notably, there was no indication that the downstream pathways involved in fatty acid synthesis or TAG storage were transcriptionally activated in the high seed lipid accumulating accession. Rather the opposite was the case, given the numerous negative correlations established between transcript abundance and flux within the plastidial (and cytosolic) fatty acid synthesis pathway (Figure 5). This finding is reminiscent of recent studies in oleaginous microalga (Li et al., 2014), and also corresponds to earlier comparative studies of plants/tissues contrasting for stored lipid accumulation (Bourgis et al., 2011; Troncoso-Ponce et al., 2011). The general implication is that the corresponding central metabolic reactions - from sucrose breakdown to TAG accumulation, and including all top TAG-responsive reactions (Schwender and Hay, 2012)—are unlikely to be controlled at the transcriptional level. Instead, post-transcriptional mechanisms might come into play. A variety of signals/metabolites (e.g., acyl-CoA, acyl carrier protein) can allosterically inhibit plastidic acetyl-CoA carboxylase and fatty acid biosynthetic enzymes, and can thereby regulate fatty acid synthesis (Andre et al., 2012). Many metabolite translocators are post-translationally modified, affecting their transport activity and eventually the provision of precursors for biosynthesis of fatty acids, amino acids, starch and other compounds (Walley et al., 2013). Allosteric feedback control is a prominent motif in the glycolytic pathway and the TCA cycle of plants (Plaxton, 1996). Metabolite-based control circuits are also common in other species including microorganisms, as recently demonstrated for *Escherichia coli* (Kochanowski et al., 2013). Tang et al. (2012) demonstrated that reactions of fatty acid biosynthesis in *Brassica* seeds have rather low metabolic control on oil accumulation.

We believe that the poor correlation between transcript data and metabolic fluxes found here for central metabolism of *B. napus* has biological significance. Similar lack of correlation has been found for prokaryotic and eukaryotic microorganisms (Daran-Lapujade et al., 2004, 2007; Chubukov et al., 2013). Despite the progress in genome scale metabolic reconstructions and next generation sequencing, there are complex functional layers between transcript and flux that make the use of transcript data to predict fluxes at best challenging (Hoppe, 2012). Central metabolism must be controlled to a large extent via post-transcriptional mechanisms. Tight transcriptional control of central metabolism might not be sufficient and/or could even be disadvantageous for an organism. In fluctuating environments (which is commonplace), input flux is continuously changing. Hence, high metabolic flexibility and fast responses are an absolute requirement for stability of vital central metabolism processes. For example, (diurnally) varying supply rates of assimilates need fast and energetically efficient means of metabolic adjustments. Fast responses are especially needed in energy metabolism, where ATP depletion would cause rapid cell death. We hypothesize that allosteric and other post-translational control mechanisms constantly re-adjust the central metabolic activities to maintain a steady state. Transcriptional control would be too imprecise and/or too slow given the fact that the peak catalytic activity of most enzymes of central metabolism is considered as being well above the necessary pathway flux (Morandini, 2009). This does not exclude that transcriptional control is involved in regulating input or output of the metabolic network which certainly can set the magnitude of flux.

In conclusion, this genome-wide study has revealed a remarkably low level of connectivity between transcript abundance and metabolic fluxes. With few exceptions (e.g., starch metabolism), *in vivo* enzyme activity/fluxes for example in glycolysis and the TCA cycle were not correlated with transcript abundance. The likelihood therefore is that fluxes in the central metabolism of the oilseed rape embryo are primarily under post-transcriptional control. Further research is clearly required to assess the contribution of translational efficiency, post-translational protein modification and allosteric control to the regulation of central metabolism pathways. Of course, it remains to be seen whether our conclusions have broader applicability beyond the biology of developing oilseeds. Recent observations in a number of microbial systems found similar discrepancies between fluxes in the central metabolism and transcript levels, and highlighted the involvement of post-translational control mechanisms (Daran-Lapujade et al., 2004, 2007; Oliveira et al., 2012; Chubukov et al., 2013). We conclude that transcript profiling can help to define stage- or tissue-specific metabolic capability, but has only limited value as an indicator of metabolic fluxes in central metabolism. This limitation needs to be borne in mind in evaluating transcriptome data, designing metabolic engineering experiments and when attempting to correlate temporal (diurnal) transcript level variation with metabolic activity. An important aim for the future should be the promotion of MFA, in particular the development of experimental protocols and tools more easily applicable to diverse plant species/organs as well as *in vivo* conditions (rather than *in vitro* approaches).

## Author contributions

Hardy Rolletschek, Ljudmilla Borisjuk and Jörg Schwender designed the research. Ljudmilla Borisjuk analyzed data. H. Roll. (seed composition) and Jörg Schwender (13C-MFA) performed research, analyzed data, and wrote the paper. Jordan O. Hay analyzed data. Christina König performed research (qPCR), analyzed data and wrote the paper. Inga Hebbelmann performed research (embryo culture, biochemical analysis). Nicolas Heinzel performed research (metabolite analysis) and analyzed data. Matthias Klapperstück analyzed data (visualization approach using VANTED) and wrote the paper. Eberhard Munz, Peter M. Jakob and Ljudmilla Borisjuk performed research (MRI) and analyzed data. Peter Denolf, Stefanie De Bodt, Henning Redestig and Evelyne Caestecker performed research (transcript analysis) and analyzed data.

### Conflict of interest statement

The authors declare that the research was conducted in the absence of any commercial or financial relationships that could be construed as a potential conflict of interest.

## Supplementary material

Supplementary Video 1 **Three-dimensional distribution of total lipid in mature seeds of oilseed rape accession 3231 (above) and 3170 (below)**. High local lipid concentrations are indicated by yellow/red color while low levels are indicated in blue. Data were acquired using magnetic resonance imaging.

Supplementary Figure 1 **Validation of DTG assignment by RNA-Seq using qPCR**. **(A)** qPCR analysis of four positive (higher abundance in accession 3170) and four negative (lower abundance in accession 3170) DTGs. **(B)** qPCR analysis of contig23563 (annotated as wrinkled1), indicating an approximately two fold higher abundance in accession 3170. Asterisks indicate statistically significant (*p* < 0.01, *t*-test) differences in abundance in the two accessions.

Supplementary Figure 2 **MapMan visualization of changes in transcription levels of genes associated with photosynthesis in the cultivated embryo of accessions 3170 and 3231**. Blue (red) denotes transcripts more (less) abundant in accession 3170.

Supplementary Figure 3 **MapMan visualization of changes in transcription levels of genes associated with the TCA cycle and the mitochondrial electron transport chain in the cultivated embryo of accessions 3170 and 3231**. Blue (red) denotes transcripts more (less) abundant in accession 3170.

Supplementary Figure 4 **Screenshot of the VANTED software**. **(A)** ViewData panel, showing Brassica transcript IDs mapped to the model reaction r35. The reaction IDs are shown, along with information regarding the congruent or opposed change for each transcript ID. **(B)** When loading Supplementary Material File 2 (frame.gml), the mapping shows an opposed fold change of transcript abundance and flux for reaction r84 (plastidial glucose-6-P/Pi antiporter), and the red frame color indicates an increased activity of this transport reaction in the accession 3170 vs. 3231.

Supplementary Table 1 **List of primers used in qPCRs**.

Supplementary Table 2 **The set of measured metabolites in the high lipid accumulator accession 3170 and the low lipid accumulator accession 3231**. Values are given in the form nmol g^−1^ dry weight, and represent the mean ± standard error (*n* = 3). Asterisks indicate statistically significant (*p* < 0.05; *t*-test) differences in abundance in the two accessions.

Supplementary Table 3 **Detailed representation of data derived from the ^**13**^C-MFA and flux variability analysis, and their relation to differentially transcribed genes (DTGs)**.

Supplementary Table 4 **Complete list of transcripts identified in the embryos of accessions 3170 and 3231**. **(A)** Normalized transcript abundances, differential fold levels and functional annotation. **(B)** GO term assignation/enrichment analysis.

Supplementary Material File 1 **Mapping_transcript_vs._flux.gml (to be used for data mapping/exploration in VANTED)**.

Supplementary Material File 2 **frame.gml (to be used for data mapping/exploration in VANTED)**.

## References

[B1] Alexa A. Rahnenführer J. Lengauer T. (2006). Improved scoring of functional groups from gene expression data by decorrelating GO graph structure. Bioinformatics 13, 1600–1607. 10.1093/bioinformatics/btl140 16606683

[B2] Allen D. K. Young J. D. (2013). Carbon and nitrogen provisions alter the metabolic flux in developing soybean embryos. Plant Physiol. 161, 1458–1475. 10.1104/pp.112.203299 23314943 PMC3585609

[B3] Alonso A. P. Goffman F. D. Ohlrogge J. B. Shachar-Hill Y. (2007). Carbon conversion efficiency and central metabolic fluxes in developing sunflower (*Helianthus annuus* L.) embryos. Plant J. 52, 296–308. 10.1111/j.1365-313X.2007.03235.x 17683473

[B5] Andre C. Haslam R. P. Shanklin J. (2012). Feedback regulation of plastidic acetyl-CoA carboxylase by 18:1-acyl carrier protein in *Brassica napus*. Proc. Natl. Acad. Sci. U.S.A. 109, 10107–10112. 10.1073/pnas.1204604109 22665812 PMC3382543

[B6] Baerenfaller K. Massonnet C. Walsh S. Baginsky S. Bühlmann P. Hennig L.. (2012). Systems-based analysis of *Arabidopsis* leaf growth reveals adaptation to water deficit. Mol. Syst. Biol. 8, 606. 10.1038/msb.2012.39 22929616 PMC3435506

[B7] Baud S. Wuillème S. To A. Rochat C. Lepiniec L. (2009). Role of WRINKLED1 in the transcriptional regulation of glycolytic and fatty acid biosynthetic genes in *Arabidopsis*. Plant J. 60, 933–947. 10.1111/j.1365-313X.2009.04011.x 19719479

[B8] Belmonte M. F. Kirkbride R. C. Stone S. L. Pelletier J. M. Bui A. Q. Yeung E. C.. (2013). Comprehensive developmental profiles of gene activity in regions and subregions of the *Arabidopsis* seed. Proc. Natl. Acad. Sci. U.S.A. 110, E435–E444. 10.1073/pnas.1222061110 23319655 PMC3562769

[B9] Benjamini Y. Hochberg Y. (1995). Controlling the false discovery rate: a practical and powerful approach to multiple testing. J. R. Stat. Soc. B Methodol. 57, 289–300.

[B10] Borisjuk L. Neuberger T. Schwender J. Heinzel N. Sunderhaus S. Fuchs J.. (2013a). Seed architecture shapes embryo metabolism in oilseed rape. Plant Cell 25, 1625–1640. 10.1105/tpc.113.111740 23709628 PMC3694696

[B11] Borisjuk L. Rolletschek H. Neuberger T. (2013b). Nuclear magnetic resonance imaging of lipid in living plants. Prog. Lipid Res. 52, 465–487. 10.1016/j.plipres.2013.05.003 23748080

[B12] Bourgis F. Kilaru A. Cao X. Ngando-Ebongue G.-F. Drira N. Ohlrogge J. B.. (2011). Comparative transcriptome and metabolite analysis of oil palm and date palm mesocarp that differ dramatically in carbon partitioning. Proc. Natl. Acad. Sci. U.S.A. 108, 12527–12532. 10.1073/pnas.1106502108 21709233 PMC3145713

[B13] Chubukov V. Uhr M. Chat L. L. Kleijn R. J. Jules M. Link H.. (2013). Transcriptional regulation is insufficient to explain substrate-induced flux changes in *Bacillus subtilis*. Mol. Syst. Biol. 9, 709. 10.1038/msb.2013.66 24281055 PMC4039378

[B14] Daran-Lapujade P. Jansen M. L. A. Daran M.-L. van Gulik W. de Winde J. H. Pronk J. T. (2004). Role of transcriptional regulation in controlling fluxes in central carbon metabolism of *Saccharomyces cerevisiae*: a chemostat culture study. J. Biol. Chem. 279, 9125–9138. 10.1074/jbc.M309578200 14630934

[B15] Daran-Lapujade P. Rossell S. Van Gulik W. M. Luttik M. A. De Groot M. J. Slijper M.. (2007). The fluxes through glycolytic enzymes in *Saccharomyces cerevisiae* are predominantly regulated at posttranscriptional levels. Proc. Natl. Acad. Sci. U.S.A. 104, 15753–15758. 10.1073/pnas.0707476104 17898166 PMC2000426

[B16] Fernie A. R. Stitt M. (2012). On the discordance of metabolomics with proteomics and transcriptomics: coping with increasing complexity in logic, chemistry, and network interactions scientific correspondence. Plant Physiol. 158, 1139–1145. 10.1104/pp.112.193235 22253257 PMC3291261

[B17] Finkemeier I. Laxa M. Miguet L. Howden A. J. M. Sweetlove L. J. (2011). Proteins of diverse function and subcellular location are lysine acetylated in *Arabidopsis*. Plant Physiol. 155, 1779–1790. 10.1104/pp.110.171595 21311031 PMC3091095

[B18] Grabherr M. G. Haas B. J. Yassour M. Levin J. Z. Thompson D. A. Amit I.. (2011). Full-length transcriptome assembly from rna-seq data without a reference genome. Nat. Biotechnol. 29, 644–652. 10.1038/nbt.1883 21572440 PMC3571712

[B20] Hay J. O. Shi H. Heinzel N. Hebbelmann I. Rolletschek H. Schwender J. (in press). Integration of a constraint-based metabolic model of *Brassica napus* developing seeds with 13C-Metabolic Flux Analysis. Front. Plant Sci. 10.3389/fpls.2014.00724 PMC4271587 25566296

[B21] Hoppe A. (2012). What mRNA abundance can tell us about metabolism. Metabolites 2, 614–631. 10.3390/metabo2030614 24957650 PMC3901220

[B22] Kochanowski K. Volkmer B. Gerosa L. Haverkorn van Rijsewijk B. R. Schmidt A. Heinemann M. (2013). Functioning of a metabolic flux sensor in *Escherichia coli*. Proc. Natl. Acad. Sci. U.S.A. 110, 1130–1135. 10.1073/pnas.1202582110 23277571 PMC3549114

[B23] Li B. Dewey C. N. (2011). Rsem: accurate transcript quantification from rna-seq data with or without a reference genome. BMC Bioinformatics 12:323. 10.1186/1471-2105-12-323 21816040 PMC3163565

[B24] Li J. Han D. Wang D. Ning K. Jia J. Wei L.. (2014). Choreography of Transcriptomes and lipidomes of nannochloropsis reveals the mechanisms of oil synthesis in microalgae. Plant Cell 26, 1645–1665. 10.1105/tpc.113.121418 24692423 PMC4036577

[B25] Livak K. J. Schmittgen T. D. (2001). Analysis of relative gene expression data using real-time quantitative PCR and the 2-ΔΔCT method. Methods 25, 402–408. 10.1006/meth.2001.1262 11846609

[B26] Maeo K. Tokuda T. Ayame A. Mitsui N. Kawai T. Tsukagoshi H.. (2009). An AP2-type transcription factor, WRINKLED1, of *Arabidopsis thaliana* binds to the AW-box sequence conserved among proximal upstream regions of genes involved in fatty acid synthesis. Plant J. 60, 476–487. 10.1111/j.1365-313X.2009.03967.x 19594710

[B27] Marillia E. F. Micallef B. J. Micallef M. Weninger A. Pedersen K. K. Zou J.. (2003). Biochemical and physiological studies of *Arabidopsis thaliana* transgenic lines with repressed expression of the mitochondrial pyruvate dehydrogenase kinase. J. Exp. Bot. 54, 259–270. 10.1093/jxb/erg020 12493853

[B28] Marmagne A. Brabant P. Thiellement H. Alix K. (2010). Analysis of gene expression in resynthesized *Brassica napus* allotetraploids: transcriptional changes do not explain differential protein regulation. New Phytol. 186, 216–227. 10.1111/j.1469-8137.2009.03139.x 20100210

[B29] Masakapalli S. K. Bryant F. M. Kruger N. J. Ratcliffe R. G. (2014). The metabolic flux phenotype of heterotrophic *Arabidopsis* cells reveals a flexible balance between the cytosolic and plastidic contributions to carbohydrate oxidation in response to phosphate limitation. Plant J. 78, 964–977. 10.1111/tpj.12522 24674596

[B30] Meyer L. J. Gao J. Xu D. Thelen J. J. (2012). Phosphoproteomic analysis of seed maturation in Arabidopsis, rapeseed, and soybean. Plant Physiol. 159, 517–528. 10.1104/pp.111.191700 22440515 PMC3375983

[B31] Morandini P. (2009). Rethinking metabolic control. Plant Sci. 176, 441–451 10.1016/j.plantsci.2009.01.005 26493133

[B32] Neuberger T. Rolletschek H. Webb A. Borisjuk L. (2009). Non-invasive mapping of lipids in plant tissue using magnetic resonance imaging. Methods Mol. Biol. 1, 485–496. 10.1007/978-1-60761-322-0_24 19763491

[B33] Oliveira A. P. Ludwig C. Picotti P. Kogadeeva M. Aebersold R. Sauer U. (2012). Regulation of yeast central metabolism by enzyme phosphorylation. Mol. Syst. Biol. 8, 623. 10.1038/msb.2012.55 23149688 PMC3531909

[B35] Piques M. Schulze W. X. Höhne M. Usadel B. Gibon Y. Rohwer J.. (2009). Ribosome and transcript copy numbers, polysome occupancy and enzyme dynamics in Arabidopsis. Mol. Syst. Biol. 5, 314. 10.1038/msb.2009.68 19888209 PMC2779082

[B36] Plaxton W. C. (1996). The organization and regulation of plant glycolysis. Ann. Rev. Plant Physiol. Plant Mol. Biol. 47, 185–214. 10.1146/annurev.arplant.47.1.185 15012287

[B37] Roberts A. Pimentel H. Trapnell C. Pachter L. (2011). Identification of novel transcripts in annotated genomes using rna-seq. Bioinformatics 27, 2325–2329. 10.1093/bioinformatics/btr355 21697122

[B38] Rohn H. Junker A. Hartmann A. Grafahrend-Belau E. Treutler H. Klapperstück M.. (2012). Vanted v2: a framework for systems biology applications. BMC Syst. Biol. 6:139. 10.1186/1752-0509-6-139 23140568 PMC3610154

[B39] Rolletschek H. Koch K. Wobus U. Borisjuk L. (2005a). Positional cues for the starch/lipid balance in maize kernels and resource partitioning to the embryo. Plant J. 42, 69–83. 10.1111/j.1365-313X.2005.02352.x 15773854

[B40] Rolletschek H. Radchuk R. Klukas C. Schreiber F. Wobus U. Borisjuk L. (2005b). Evidence of a key role for photosynthetic oxygen release in oil storage in developing soybean seeds. New Phytol. 167, 777–786. 10.1111/j.1469-8137.2005.01473.x 16101914

[B41] Rontein D. Dieuaide-Noubhani M. Dufourc E. J. Raymond P. Rolin D. (2002). The metabolic architecture of plant cells. J. Biol. Chem. 277, 43948–43960. 10.1074/jbc.M206366200 12226084

[B42] Schwender J. (2008). Metabolic flux analysis as a tool in metabolic engineering of plants. Curr. Opin. Biotechnol. 19, 131–137. 10.1016/j.copbio.2008.02.006 18378441

[B43] Schwender J. Goffman F. Ohlrogge J. B. Shachar-Hill Y. (2004). Rubisco without the Calvin cycle improves the carbon efficiency of developing green seeds. Nature 432, 779–782. 10.1038/nature03145 15592419

[B44] Schwender J. Hay J. O. (2012). Predictive modeling of biomass component tradeoffs in *brassica napus* developing oilseeds based on *in silico* manipulation of storage metabolism. Plant Physiol. 160, 1218–1236. 10.1104/pp.112.203927 22984123 PMC3490581

[B45] Schwender J. Shachar-Hill Y. Ohlrogge J. B. (2006). Mitochondrial metabolism in developing embryos of *Brassica napus*. J. Biol. Chem. 281, 34040–34047. 10.1074/jbc.M606266200 16971389

[B46] Spielbauer G. Margl L. Hannah L. C. Römisch W. Ettenhuber C. Bacher A.. (2006). Robustness of central carbohydrate metabolism in developing maize kernels. Phytochemistry 67, 1460–1475. 10.1016/j.phytochem.2006.05.035 16815503

[B47] Sweetlove L. J. Obata T. Fernie A. R. (2014). Systems analysis of metabolic phenotypes: what have we learnt? Trends Plant Sci. 19, 222–230. 10.1016/j.tplants.2013.09.005 24139444

[B48] Tang M. Guschina I. A. O'Hara P. Slabas A. R. Quant P. A. Fawcett T.. (2012). Metabolic control analysis of developing oilseed rape (*Brassica napus* cv Westar) embryos shows that lipid assembly exerts significant control over oil accumulation. New Phytol. 196, 414–426. 10.1111/j.1469-8137.2012.04262.x 22901003

[B49] Thimm O. Bläsing O. Gibon Y. Nagel A. Meyer S. Krüger P.. (2004). MAPMAN: a user-driven tool to display genomics data sets onto diagrams of metabolic pathways and other biological processes. Plant J. 37, 914–939. 10.1111/j.1365-313X.2004.02016.x 14996223

[B50] Trapnell C. Pachter L. Salzberg S. L. (2009). Tophat: discovering splice junctions with rna-seq. Bioinformatics 25, 1105–1111. 10.1093/bioinformatics/btp120 19289445 PMC2672628

[B51] Troncoso-Ponce M. A. Kilaru A. Cao X. Durrett T. P. Fan J. Jensen J. K.. (2011). Comparative deep transcriptional profiling of four developing oilseeds. Plant J. 68, 1014–1027. 10.1111/j.1365-313X.2011.04751.x 21851431 PMC3507003

[B52] Walley J. W. Shen Z. Sartor R. Wu K. J. Osborn J. Smith L. G.. (2013). Reconstruction of protein networks from an atlas of maize seed proteotypes. Proc. Natl. Acad. Sci. U.S.A. 110, E4808–E4817. 10.1073/pnas.1319113110 24248366 PMC3856832

[B53] Williams T. C. R. Miguet L. Masakapalli S. K. Kruger N. J. Sweetlove L. J. Ratcliffe R. G. (2008). Metabolic network fluxes in heterotrophic *Arabidopsis* cells: stability of the flux distribution under different oxygenation conditions. Plant Physiol. 148, 704–718. 10.1104/pp.108.125195 18667721 PMC2556809

[B54] Williams T. C. R. Poolman M. G. Howden A. J. M. Schwarzlander M. Fell D. A. Ratcliffe R. G.. (2010). A genome-scale metabolic model accurately predicts fluxes in central carbon metabolism under stress conditions. Plant Physiol. 154, 311–323. 10.1104/pp.110.158535 20605915 PMC2938150

