# Supplementary figures and images for: Transcript abundance on its own cannot be used to infer fluxes in central metabolism
Source: Front Plant Sci. 2014 Nov 28;5:668. doi: 10.3389/fpls.2014.00668 (PMC4246676; doi:10.3389/fpls.2014.00668)

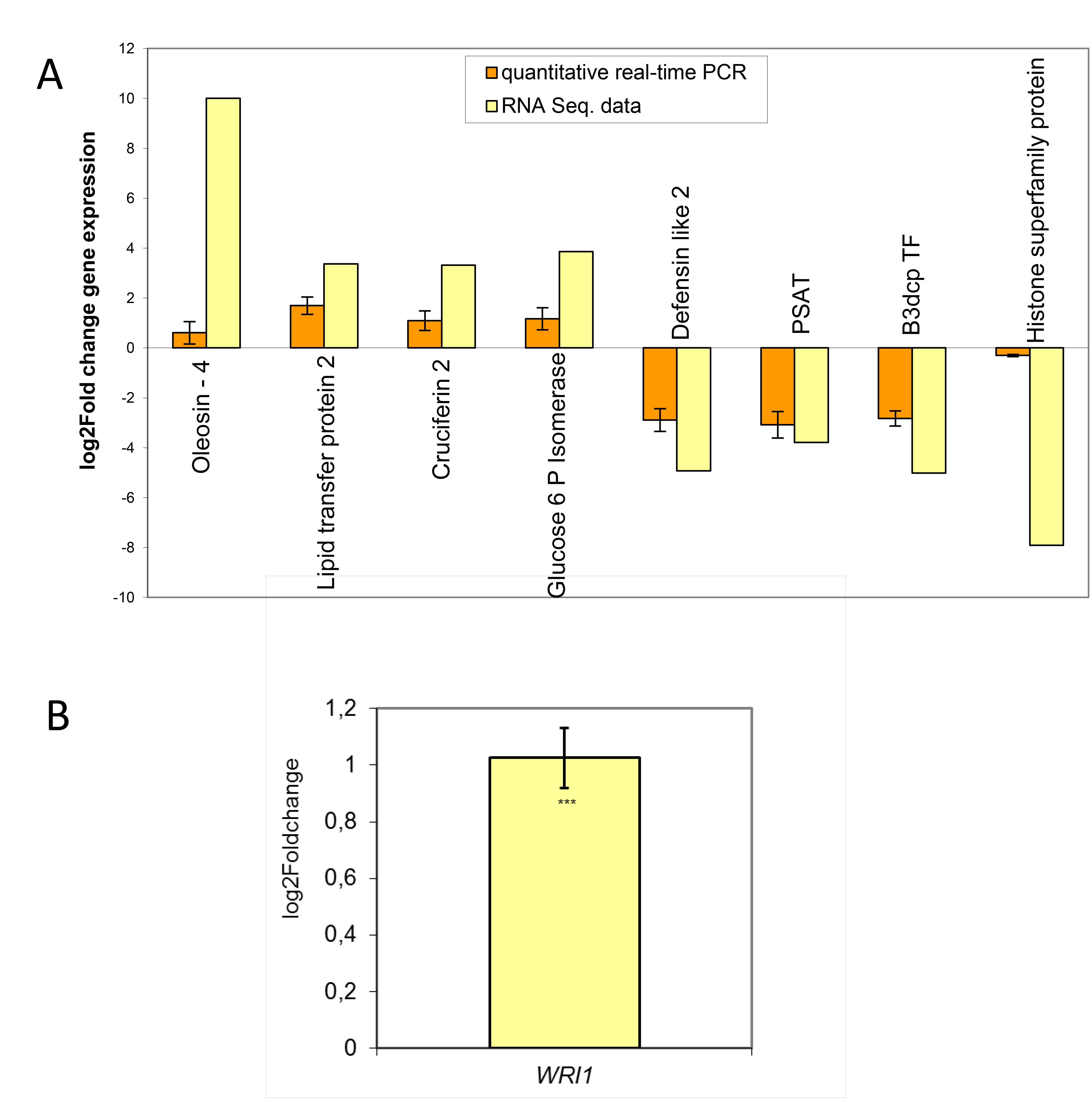

Supplement: Supplementary Figure 1 — Validation of DTG assignment by RNA-Seq using qPCR. (A) qPCR analysis of four positive (higher abundance in accession 3170) and four negative (lower abundance in accession 3170) DTGs. (B) qPCR analysis of contig23563 (annotated as wrinkled1), indicating an approximately two fold higher abundance in accession 3170. Asterisks indicate statistically significant (p < 0.01, t-test) differences in abundance in the two accessions. [file Image1.JPEG]

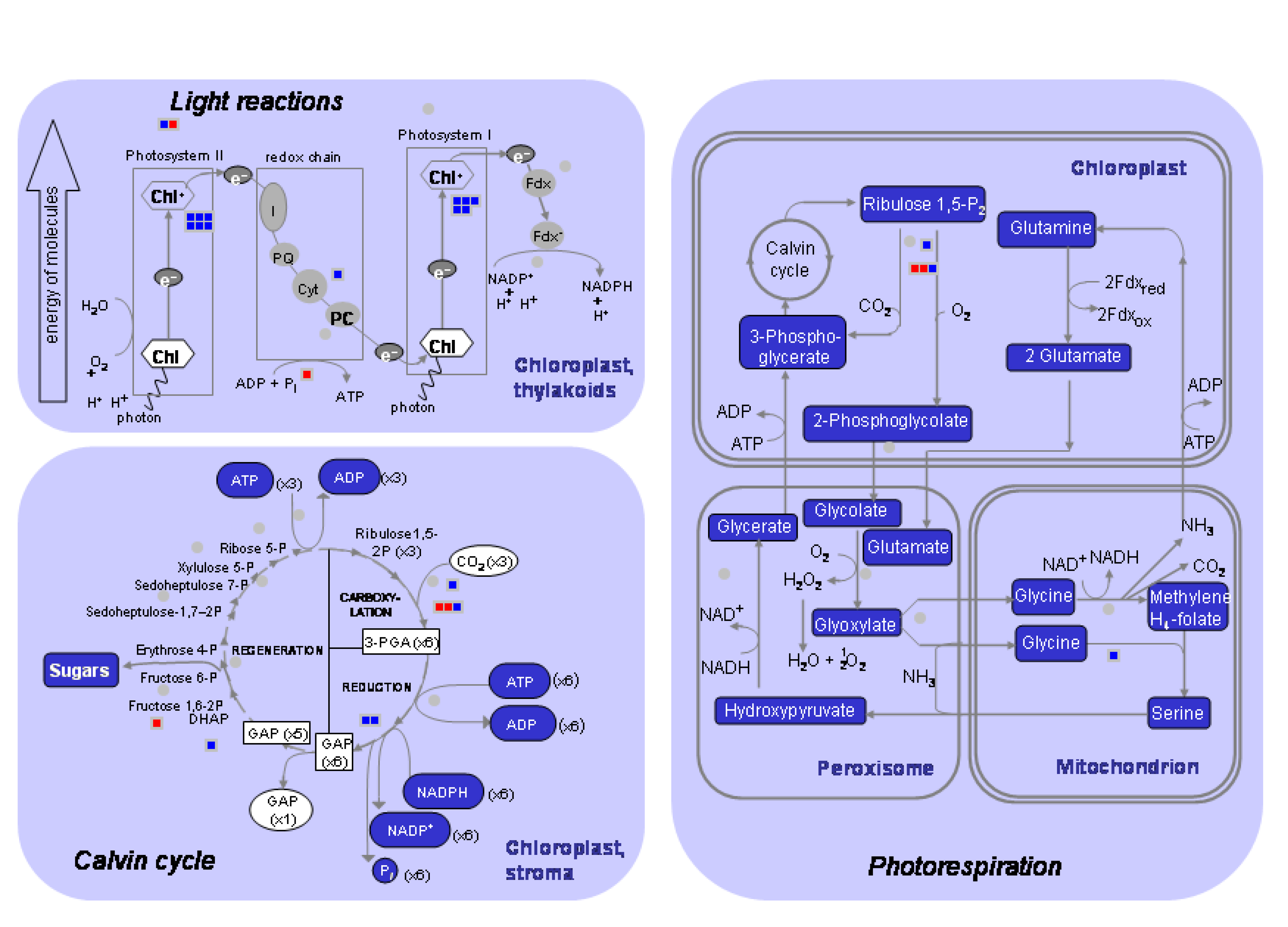

Supplement: Supplementary Figure 2 — MapMan visualization of changes in transcription levels of genes associated with photosynthesis in the cultivated embryo of accessions 3170 and 3231. Blue (red) denotes transcripts more (less) abundant in accession 3170. [file Image2.JPEG]

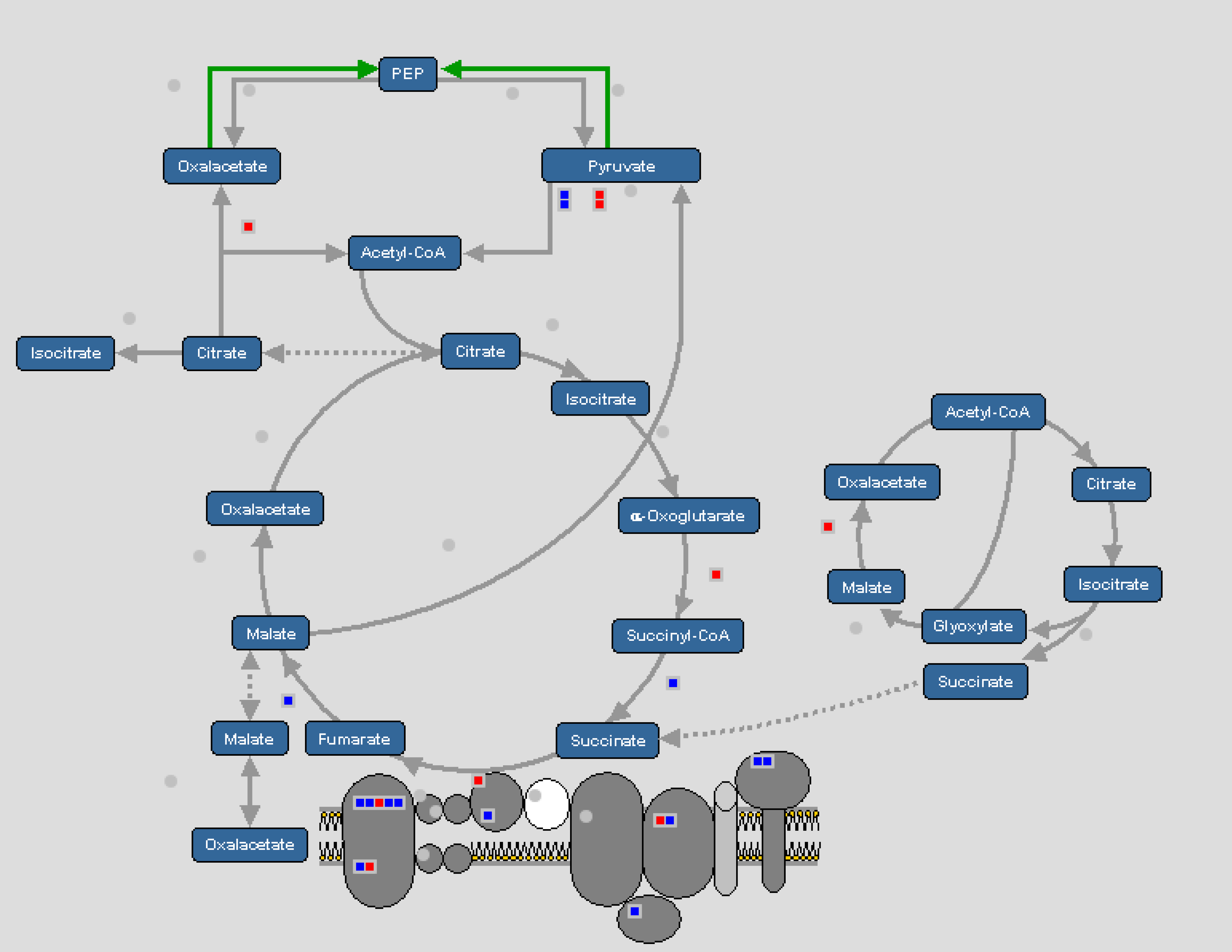

Supplement: Supplementary Figure 3 — MapMan visualization of changes in transcription levels of genes associated with the TCA cycle and the mitochondrial electron transport chain in the cultivated embryo of accessions 3170 and 3231. Blue (red) denotes transcripts more (less) abundant in accession 3170. [file Image3.JPEG]

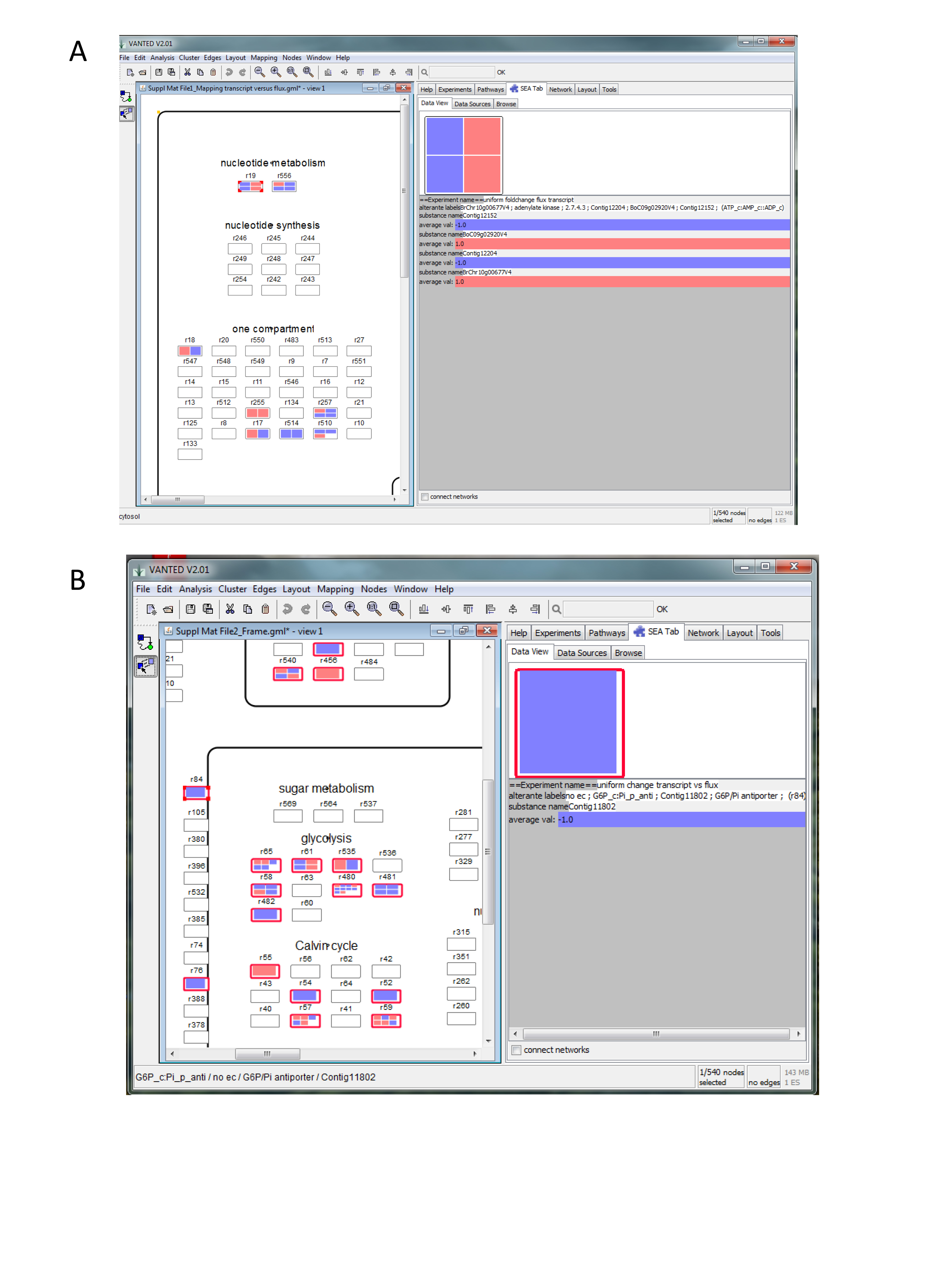

Supplement: Supplementary Figure 4 — Screenshot of the VANTED software. (A) ViewData panel, showing Brassica transcript IDs mapped to the model reaction r35. The reaction IDs are shown, along with information regarding the congruent or opposed change for each transcript ID. (B) When loading Supplementary Material File 2 (frame.gml), the mapping shows an opposed fold change of transcript abundance and flux for reaction r84 (plastidial glucose-6-P/Pi antiporter), and the red frame color indicates an increased activity of this transport reaction in the accession 3170 vs. 3231. [file Image4.JPEG]
