# Supplementary material for: Transcript abundance on its own cannot be used to infer fluxes in central metabolism
Source: Front Plant Sci. 2014 Nov 28;5:668. doi: 10.3389/fpls.2014.00668 (PMC4246676; doi:10.3389/fpls.2014.00668)
Supplement: Supplementary Table 1 — List of primers used in qPCRs. [file Table1.PDF]

Supplemental Table 1. List of primers used in qPCR

Contig	Primer Name	5'-->3' Primer Sequence
Contig14619	Oleosin S2-2 fwd 2	TACCTGGCTCAGTGGTCTCA
Contig14619	Oleosin S2-2 rev 2	CGAGTGATACGGTTCCGGAG
Contig34414	NLTP2 fwd 1	CGGGCCAGGTTGTTTAGACT
Contig34414	NLTP2 rev 1	GTTGGCATGCTTGGTCTTGG
Contig255	Cru4 fwd 2	CAAGGTCAGGGTCAAGGCAA
Contig255	Cru4 rev 2	AACCATTGAGCTACACCGGG
BrChr9g01612.01V4	G6PI fwd 2	TGGGGAGTTGAGCTAGGGAA
BrChr9g01612.01V4	G6PI rev 2	ACTTCACATCTTCGGCTCGG
BrChr5g02946.01V4	DEF2 fwd 2	GCGACTACTGCAGCGACATA
BrChr5g02946.01V4	DEF2 rev 2	GAGCCTCATTACTTCGGGCT
Contig6843	SERC_ARATH fwd 3	TGATGGAGAGGGGTTTGGTG
Contig6843	SERC_ARATH rev 3	ATCCCTCTCCCACCAACTCT
Contig10069	B3dcp fwd 1	ACCTGATCACCGGTTCGTTT
Contig10069	B3dcp rev 1	AAAACCAGAGCCAGAACCGG
Contig4914	H2B.11 fwd 1	TTAGTCACGGCCTTGGTTCC
Contig4914	H2B.11 rev 1	GAACAGCTTCATCAACGCCA
Contig23563	WRI1 1 fwd	CCTGTGAGCCAAGCCAATCA
Contig23563	WRI1 1 rev	CAATGCAGCAAGTGACCACC
Contig4947	CSK fwd 2	CATCGTCTTCCTCCTCGCAA
Contig4947	CSK rev 2	GGATTGGGTTGGGGAAGAGG
